# Facile synthesis, characterization and enhanced catalytic reduction of 4-nitrophenol using NaBH_4_ by undoped and Sm^3+^, Gd^3+^, Hf^3+^ doped La_2_O_3_ nanoparticles

**DOI:** 10.1186/s40580-019-0181-6

**Published:** 2019-04-10

**Authors:** Guguloth Ravi, Madderla Sarasija, Dasari Ayodhya, Lunavath Shanthi Kumari, Dongamanti Ashok

**Affiliations:** 1 Department of Chemistry, Osmania University, Hyderabad, TS 500007 India; 2 Department of Chemistry, Satavahana University, Karimnagar, TS 505002 India; 3 Department of BioChemistry, Osmania University, Hyderabad, TS 500007 India

**Keywords:** Facile synthesis, La_2_O_3_ NPs, Dopants, Catalytic activity, 4-Nitrophenol reduction, Kinetics

## Abstract

This work focuses on the synthesis of undoped and doped lanthanum oxide nanoparticles (La_2_O_3_ NPs) by a simple co-precipitation method for the catalytic reduction of 4-nitrophenol (4-NP) using NaBH_4_ as a reducing agent. Their optical properties, morphologies, structure, chemical compositions and electronic properties were carefully characterized by XRD, FTIR, SEM, TEM, PL and UV–visible absorption spectroscopy. The SEM and TEM images showed various shape morphologies and sizes of the particles. The XRD pattern revealed a polycrystalline nature with the hexagonal structure of the La_2_O_3_ NPs. The synthesized undoped and doped La_2_O_3_ NPs were also employed as catalysts for the reduction of 4-nitrophenol, it shows that the doped (Sm^3+^, Gd^3+^ and Hf^3+^) La_2_O_3_ NPs provided better catalytic activity than the undoped La_2_O_3_ NPs. Moreover, Hf^3+^ doped La_2_O_3_ NPs exhibited an enhanced catalytic activity for the reduction of 4-nitrophenol to 4-aminophenol in 90 min. The catalytic conversion was studied by UV–vis spectroscopy with high reduction rate (k = 2.048 min^−1^). The applications of the present study may utilize in the removal of toxic pollutants in a cleaning of environmental pollution as well as in industrial applications.

## Introduction

In the past few years, due to the concerns about environmental pollution and potential exhaustion possibility of fossil fuels, more and more attention has been paid to the development of green and renewed energy resources. Therefore, the research on materials is necessary for the prevention of environmental pollution by the degradation or conversion of toxic compounds (dyes, pesticides and chlorinated phenols such as 4-nitrophenol) into the non-polluting compounds. 4-Nitrophenol (4-NP) has been wildly used in several applications such as pharmaceutical, dyeing agent, plastics, pesticides and anti-corrosion lubricant [1–3]. This compound has been identified as one of the most hazardous and toxic pollutants generated mainly from agricultural and industrial sources [4, 5]. Therefore, several methods have been introduced to remove 4-NP from wastewater with various advantages and limitations such as adsorption [6], microbial degradation [7], electrocoagulation [8] and reduction [9]. Moreover, apart from the industrial and environmental viewpoints, reduction of 4-nitrophenol is considered as a model reaction for catalytic study [2, 10]. It is well-known that without a catalyst, the reduction of- 4-NP is extremely slow. Therefore, many investigators have paid attention to the development of catalysts for the reduction reaction of 4-NP.

The synthesis, production and manipulation of materials on the nanoscale are currently one of the favorable areas of research which also attracts the industrialists for designing and fabricating new functional materials with novel special properties [11, 12]. Rare earth elements are attractive materials for industry and play an important role in a number of current technologies as active components. La_2_O_3_ is a rare earth metal oxide, which has a band gap of 4.3 eV and the lowest lattice energy with the high electric constant. La_2_O_3_ ultrafine powders have a lot of attractive properties for industrial and technological applications. The electronic and magnetic properties of La_2_O_3_ differ considerably from those of the other oxides in the series because La^3+^ is the only trivalent rare earth cation that lacks 4f electrons and has the simple Xenon electronic structure. Because of their unique electronic configuration [4f electrons] lanthanides have been applied in various fields; also these lanthanide-based materials have attractive and interesting magnetic [13], optical [14, 15], electrical and therapeutic [16] properties.

Among the lanthanides, lanthanum has been extensively examined for its unique properties [17]. The lanthanum based materials have been synthesized in various compositions such as La(OH)_3_ [18], LaF_3_ [15], La_2_(CO_3_)_3_ [19], LaPO_4_ [20], LaBO_3_ [21], LaOF [22], La_2_Sn_2_O_7_ [23], La_2_O_3_ [24] nanoparticles. Although many methods have been developed for the synthesis of lanthanum nanostructures including hydrothermal [25], solvothermal [26], microemulsion or reverse micelles [27], sol–gel [28], laser deposition [29] and other chemical and physical methods; but some of these methods are affected by long reaction time, high temperature, high pressure, expensive surface materials and so on. Based on electronic, optical, and chemical characteristics arising from lanthanides 4f electrons, lanthanide compounds have been widely used as high-performance luminescent devices, upconversion materials, catalysts, and time-resolved fluorescence (TRF) labels for biological detection [30, 31]. In particular, recently La_2_O_3_ NPs were used in catalytic applications as a promising catalyst for the catalytic oxidative cracking of *n*-propane [32], ethanol oxidation [33], and degradation of rhodamine B under visible light irradiation [34]. From the inspiration of these studies, we have investigated the reduction of 4-NP to 4-AP using La_2_O_3_ NPs and NaBH_4_ as a reducing agent.

In this work, we report the undoped and doped La_2_O_3_ NPs were successfully prepared from the reaction of lanthanum nitrate and urea by a simple co-precipitation method. The prepared products were characterized by XRD, SEM, TEM, UV–visible absorption spectroscopy, PL and FT-IR spectroscopy. The effect of La_2_O_3_ NPs in the presence and absence of dopants has been investigated in the catalytic reduction of 4-NP to 4-AP using NaBH_4_ as a reducing agent. It was found that the presence of dopants significantly improved the catalytic performance of La_2_O_3_ NPs in the 4-NP conversion catalyzed by sodium borohydride (NaBH_4_) as a strong reducing agent than the undoped La_2_O_3_ NPs. The application of the present work may utilize in the removal of industrial pollutants for the prevention of environmental pollution.

## Experimental

### Materials and methods

All the materials were obtained from commercial suppliers and were used without further purification. Lanthanum nitrate hexahydrate (La(NO_3_)_3_·6H_2_O), carbamide (CH_4_N_2_O) and NaBH_4_ were purchased from Merck Chemicals, India. Samarium nitrate, gadolinium nitrate hexahydrate and hafnium nitrate were purchased from S D Fine Chemicals, India. The double distilled water used as a solvent for the preparation of stock solutions.

### Synthesis of undoped La_2_O_3_ nanoparticles

The undoped La_2_O_3_ NPs has been synthesized by a simple co-precipitation method [35] using lanthanum nitrate and urea as starting materials. In a typical synthesis, 0.05 M of lanthanum nitrate and 0.05 M of urea were dissolved in 100 ml of double distilled water. The precursor solution was transferred into a round bottom flask and maintained at a constant temperature of 60 °C for 12 h. Then, the mixture was stirred for 30 min under the magnetic stirring for uniform distribution and formation of nanoparticles. The final products were collected by ultra-centrifugation and washed the obtained precipitate several times with ethanol and double distilled water for removal of unreacted precursors. Finally, the prepared La_2_O_3_ NPs were calcinated at 500 °C for 1 h and purified samples were further used in applications.

### Synthesis of Sm^3+^, Gd^3+^ and Hf^3+^ doped La_2_O_3_ nanoparticles

The Sm^3+^, Gd^3+^ and Hf^3+^ doped La_2_O_3_ NPs have been synthesized by a simple co-precipitation method [35]. In a typical synthesis, 0.05 M of lanthanum nitrate hexahydrate, 0.001 M metal salts (samarium nitrate for Sm^3+^; gadolinium nitrate hexahydrate for Gd^3+^ and hafnium nitrate for Hf^3+^) and appropriate concentration of urea were dissolved in 100 ml double distilled water. The precursor solution was transferred into a round bottom flask and maintained at a constant temperature of 60 °C for 12 h. Then, the mixture was stirred for 30 min under the magnetic stirring for uniform distribution and formation of nanoparticles. The final products were collected by ultra-centrifugation and washed the obtained precipitate several times with ethanol and double distilled water for removal of unreacted precursors. Finally, the as-prepared Sm^3+^, Gd^3+^ and Hf^3+^ doped La_2_O_3_ NPs were calcinated at 500 °C for 1 h. The collected samples were characterized by various physicochemical techniques for the confirmation of La_2_O_3_ NPs.

### Characterizations

The synthesized undoped and doped (Sm^3+^, Gd^3+^ and Hf^3+^) La_2_O_3_ NPs, were analyzed by various physicochemical techniques. The UV–vis spectroscopic measurements were made at room temperature using a Shimadzu UV-3600 model double beam UV–vis spectrophotometer in the range of wavelength 200–800 nm. Fourier transform infrared (FTIR) spectra of La_2_O_3_ NPs were recorded in KBr pellets using an FTIR spectrophotometer (Bruker Optics, Germany, Model no. Tensor 27) in the range of wavenumber 400–4000 cm^−1^. X-ray diffraction (XRD) measurement was carried out on X’pert Pro X-ray diffractometer (Panalytical B.V., The Netherlands) operating at 40 kV and a current of 30 mA at a scan rate of 0.388 min^−1^. The morphology of the doped and undoped La_2_O_3_ NPs was characterized by scanning electron microscopy (SEM, ZEISS EVO18, 15 kV). The size distribution and crystallinity of the synthesized samples were obtained by transmission electron microscopy (TEM) measurement, casting NPs dispersion on carbon-coated copper grids and allowing drying at room temperature. TEM measurements were done on Tecnai G2 FEI F12 operated at an accelerating voltage of 200 kV.

### Catalytic reduction of 4-nitrophenol

The catalytic reduction process of 4-NP was monitored by UV–vis absorption spectra. In a typical process, 0.5 ml of 0.15 M freshly prepared NaBH_4_ solution was added to a solution containing 0.05 ml of 0.005 M 4-NP and 2.25 ml of deionized water. At this stage, the 4-NP was converted into 4-nitrophenolate anion. After that, the 10 mg of catalyst (undoped and doped La_2_O_3_ NPs) was added and the reaction was spectrophotometrically monitored at different time intervals. A gradual change of the solution color from bright yellow to colorless was observed during the reaction. After reaction, the catalyst was recovered by precipitation/centrifugation. The absorption spectra were recorded within the wavelength range of 250–500 nm. The rate constants of the reduction reaction were calculated by measuring the peak intensity evolution every minute at wavelengths of 400 nm for 4-NP. The investigation of catalytic conversion was also studied using undoped, Sm^3+^, Gd^3+^ and Hf^3+^ doped La_2_O_3_ NPs against the reaction time.

## Results and discussion

### UV–vis absorption analysis

**Fig. 1:**
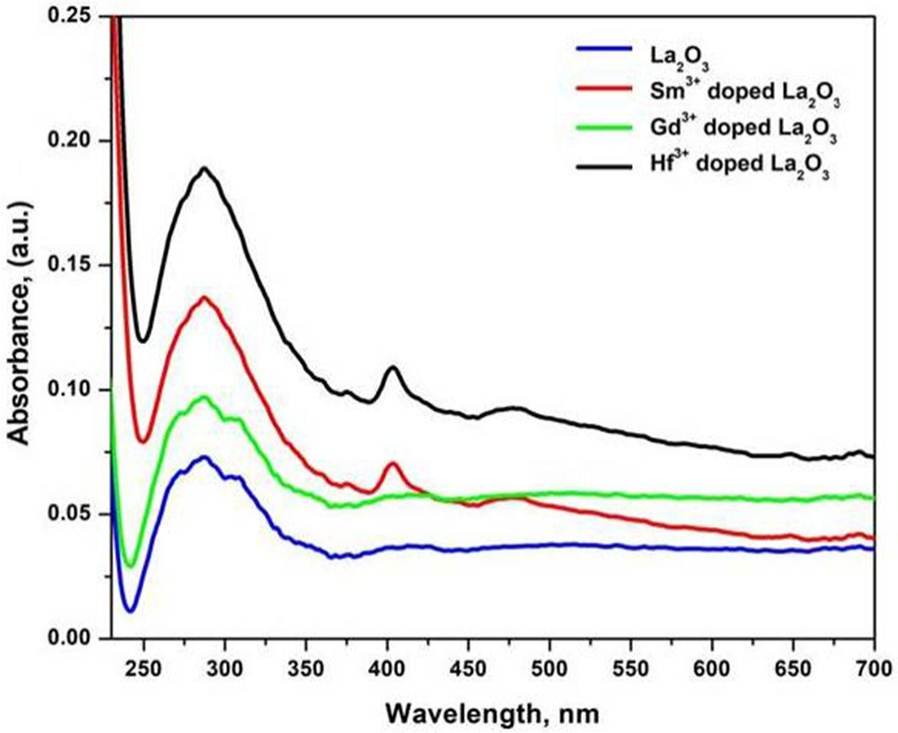
Room temperature optical absorption spectrum of undoped and doped La_2_O_3_ nanoparticles

UV–visible absorption measurement is one of the most important methods to reveal the optical properties of synthesized La_2_O_3_ NPs samples. The optical absorbance spectrum of La_2_O_3_ NPs for the wavelength length range (200–700 nm) was recorded using a UV–visible spectrophotometer. Figure 1 shows the UV absorption spectrum of as-prepared undoped and doped (Sm^3+^, Gd^3+^ and Hf^3+^) La_2_O_3_ NPs prepared by a simple co-precipitation method. The maximum absorption of undoped, Sm^3+^, Gd^3+^ and Hf^3+^ doped La_2_O_3_ NPs occurred at 250–310 nm. The doped La_2_O_3_ NPs showed high intensity and a blue shift from the undoped La_2_O_3_ NPs due to the quantum confinement. The bandgap values are calculated from the Planks equation: E_g_ = 1260/λ, where E_g_ is the band gap and λ is the wavelength of the sample. The approximate band gap values of the prepared undoped, Sm^3+^, Gd^3+^ and Hf^3+^ doped La_2_O_3_ NPs samples as calculated from the absorption spectrum are 5.24, 5.36, 5.51, and 5.74 eV, respectively. It is confirmed that the doped La_2_O_3_ NPs exhibited higher band gap and smaller in size due to the quantum confinement effect [36].

### Photoluminescence study

**Fig. 2:**
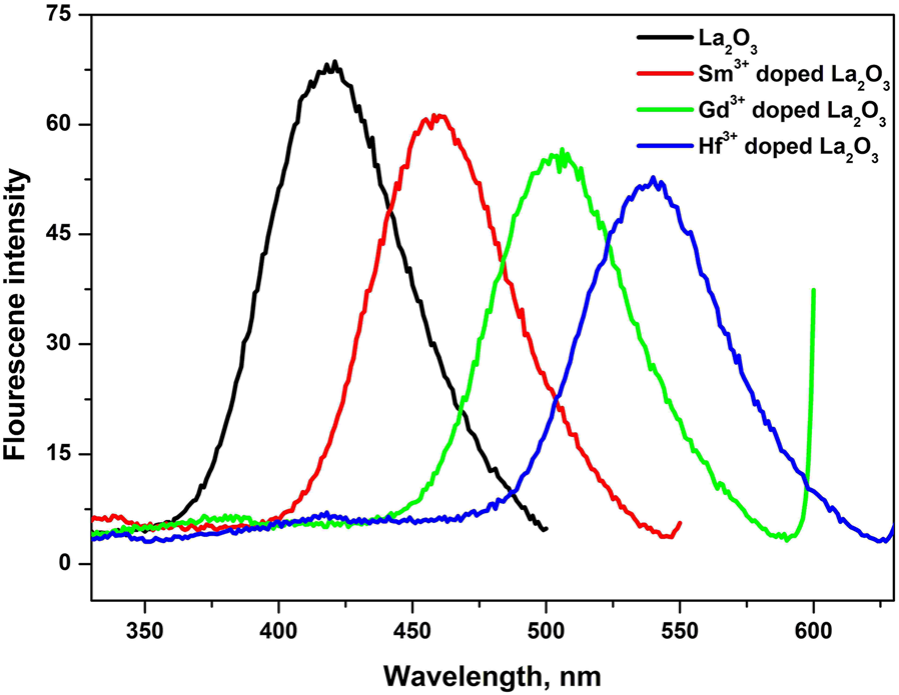
Room temperature optical PL spectrum of undoped and doped La_2_O_3_ nanoparticles

The photoluminescence (PL) is one of the significant techniques which reach more information regarding defects and impurities on the energy states inside oxide film. PL measurements were performed in the wavelength ranging from 200 to 900 nm using 350 nm as excitation wavelength. Figure 2 shows that PL spectra of undoped and Sm^3+^, Gd^3+^ and Hf^3+^ doped La_2_O_3_ at room temperature. It exhibits the identical emission peaks at 435, 473, 511 and 545 nm of undoped and Sm^3+^, Gd^3+^ and Hf^3+^ doped La_2_O_3_ respectively. All these emissions are attributed to deep levels and states localized in the band gap of La_2_O_3_ [37]. Particularly, green emission peaks were observed around 472.5, 509.40 and 544.35 nm. This emission has been attributed to singly ionized oxygen vacancies in La_2_O_3_, and the emission results from the radioactive recombination of a photogenerated hole with an electron occupying an oxygen vacancy, which is commonly referred to as the green luminescence mechanism of an important precursor to lanthanum oxide.

### FTIR analysis

**Fig. 3:**
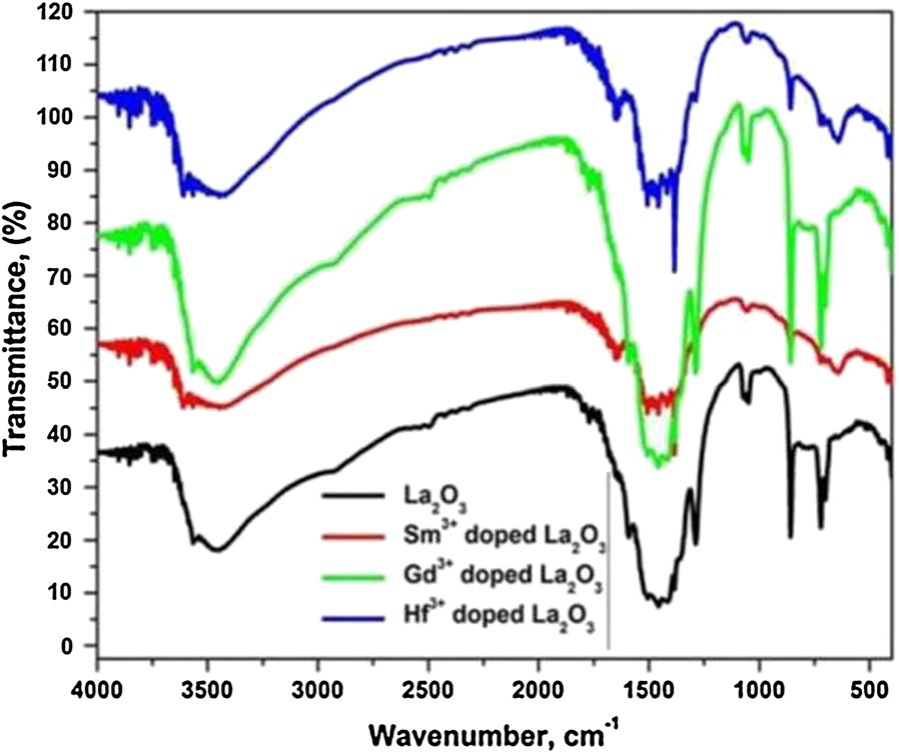
FTIR spectrum of the doped and undoped La_2_O_3_ nanoparticles

The FTIR spectra have been recorded for the as-prepared undoped and doped (Sm^3+^, Gd^3+^ and Hf^3+^) La_2_O_3_ samples in the wave number range from 500 to 4000 cm^−1^. The samples have been admixed with KBr, thoroughly mixed and pelletized by pressing under sufficient pressure, before FTIR analysis. From all FTIR spectra, the frequency of various functional groups present in the prepared sample can be predicted with the position of the vibration peaks. The FTIR spectra of doped and undoped La_2_O_3_ NPs exhibited similar absorption peaks but the change in the position of wavenumbers due to the interaction of different metal bonds in the doped samples. The broad absorption bands at 3600–3400 cm^−1^ are assigned to O–H stretching vibration of water molecules, it shows the presence of moisture in the sample. A sharp peak was observed at about 1488 cm^−1^ which can be attributed to stretching vibration of C–C and medium absorption bands at 1302 and 1077 cm^−1^ are possibly due to stretching vibrations of C=O bonding and it may appear from the absorption of atmospheric CO_2_. The strong vibration band observed at 845 cm^−1^ is ascribed to the stretching vibrations of ions in the La_2_O_3_ NPs. The medium to strong absorption bands at 643 cm^−1^ was because of La–O stretching and it may existence of the presence of La_2_O_3_. The strong and sharp peaks in FT-IR result reveals that crystalline La_2_O_3_ is obtained and it is well in agreement with the result of XRD (Fig. 3).

### XRD analysis

**Fig. 4:**
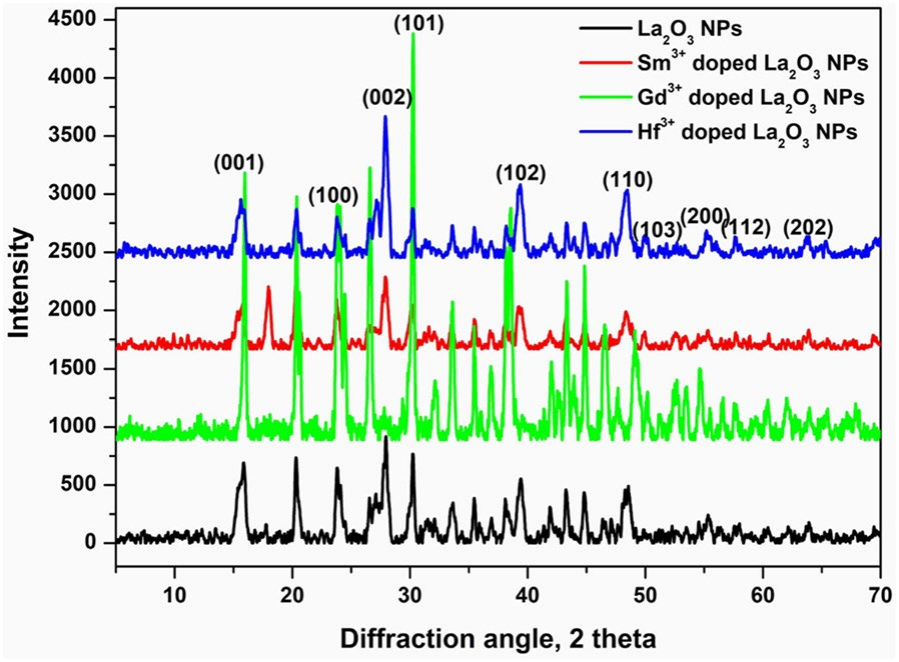
X-ray diffraction patterns for doped and undoped La_2_O_3_ nanoparticles

The crystal structure of the undoped and doped (Sm^3+^, Gd^3+^ and Hf^3+^) La_2_O_3_ NPs were characterized by XRD in the diffraction angle (2θ) range between 10° and 80° and shown in Fig. 4. It displays the crystalline structure and phase purity of the as-prepared La_2_O_3_ NPs; it can be concluded from the appearance of more than one prominent peak that the prepared La_2_O_3_ samples are polycrystalline in nature. The obtained XRD characteristics peaks shows that the maximum intensity peak was observed at (0 0 1), (1 0 0), (0 0 2), (1 0 1), (1 0 2), (1 1 0), (1 0 3), (2 0 0), (1 1 2) and (2 0 2) crystal planes at 16.1°, 26.2°, 29.7°, 30.4°, 39.8°, 46.8°, 52.8°, 54.1°, 56.2° and 63.1° diffraction angles, respectively. The well-extended intensity peaks indicate the polycrystalline nature and size reduction of La_2_O_3_ NPs. All diffraction peaks can be indexed to the hexagonal structure (space group P3m1 with cell constant a = 3.9381 Å, b = 3.9381 Å and c = 6.1361 Å, JCPDS No.: 50-0602) and comparable with an earlier report [38]. The broadening of the peaks indicated that the particles were of nanometer scale. The crystal structures of the samples all crystallized in pure hexagonal La_2_O_3_ phase and no additional diffraction peaks were formed, indicating that the Sm^3+^, Gd^3+^, and Hf^3+^ ions are efficiently dissolved in the La_2_O_3_ host lattice by replacing the La^3+^. The average crystallite sizes of the synthesized doped and undoped La_2_O_3_ NPs were calculated from the XRD patterns using Debye–Scherrer formula:

$$D = \frac{K \lambda }{\beta \cos \theta }$$

where K is a constant (0.89), λ is the wavelength of X-ray (λ = 1.5418 A_0_), θ is the diffraction angle for the peak and β is full width at half maximum (FWHM). The observed sizes were 22 nm, 15 nm, 12 nm and 11 nm for as-prepared undoped, Sm^3+^, Gd^3+^ and Hf^3+^ doped La_2_O_3_ NPs, respectively. From the tiny size of the samples may give better catalytic activities than the bulk size of the samples. It suggested that the doped La_2_O_3_ NPs exhibited better catalytic activity than the undoped La_2_O_3_ NPs.

### Morphology analysis

**Fig. 5:**
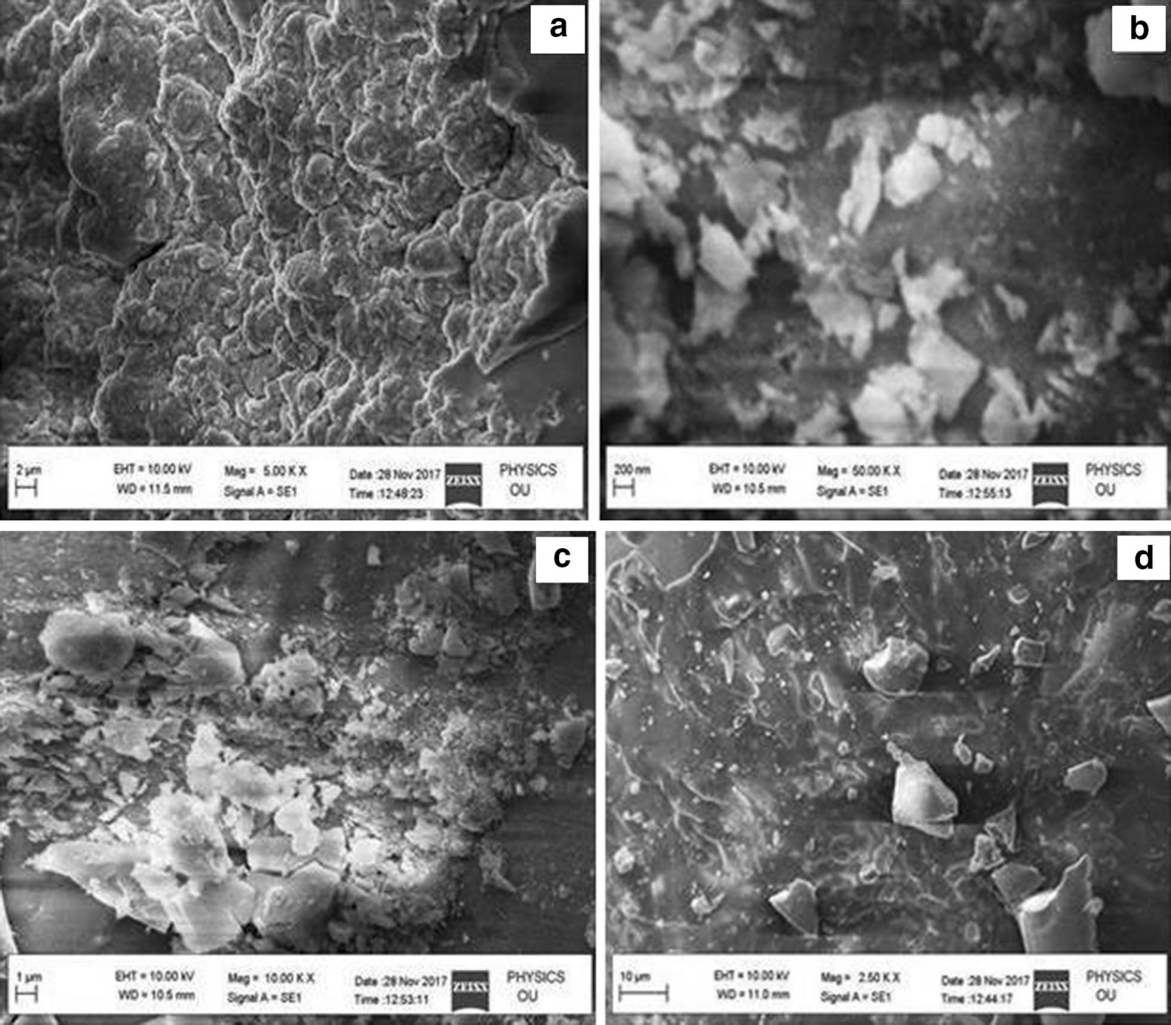
**a**–**d** SEM images of undoped and doped La_2_O_3_ nanoparticles

**Fig. 6:**
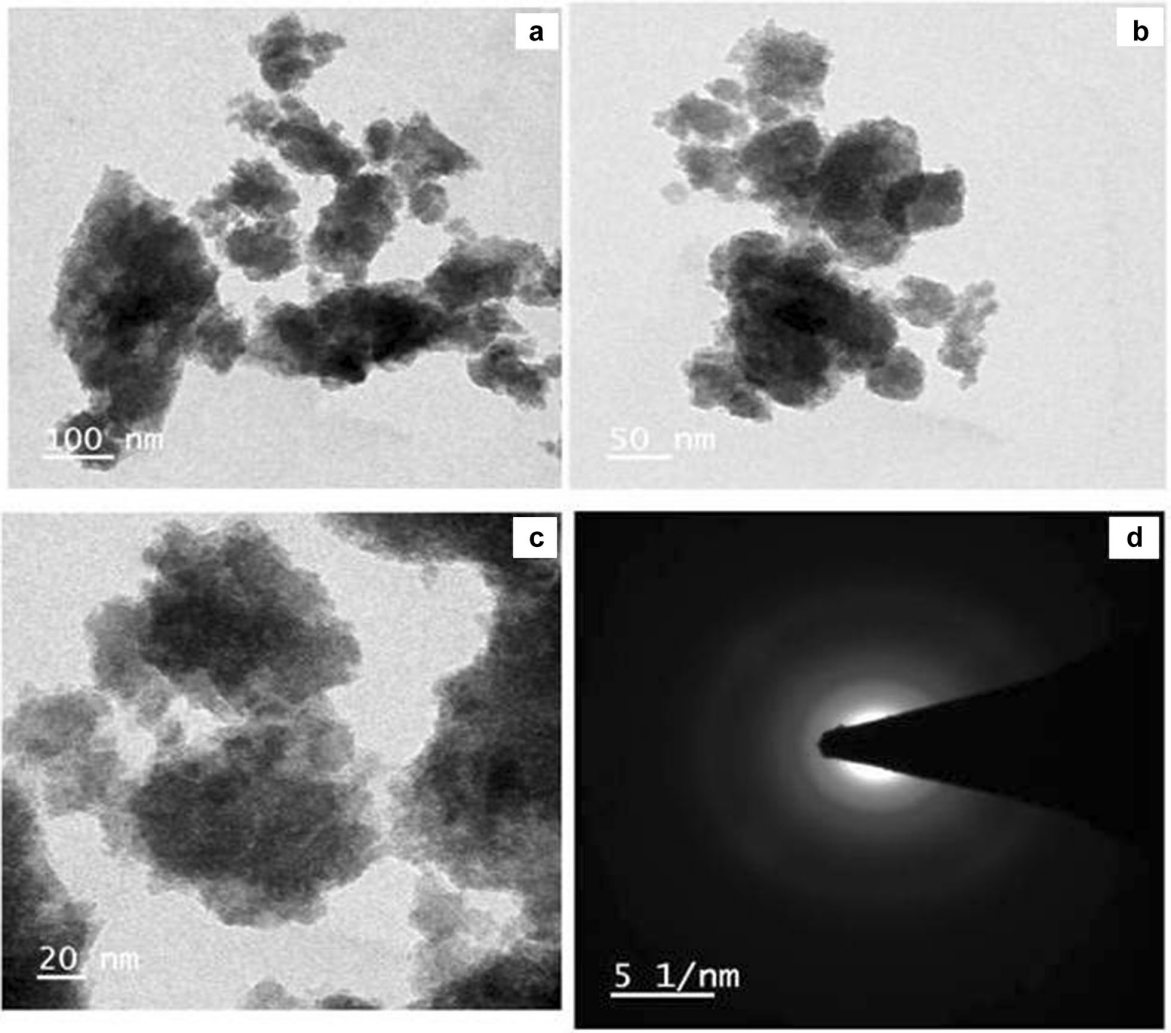
**a**–**c** TEM images of doped La_2_O_3_ NPs and **d** its SAED pattern

The microstructure and morphology of the synthesized undoped and doped (Sm^3+^, Gd^3+^ and Hf^3+^) La_2_O_3_ NPs were observed using SEM and TEM with different magnifications and shown in Figs. 5a–d and 6a–d. The SEM images reveal that different morphologies of La_2_O_3_ samples and sizes are about with micrometer range. Apart from this, some particle agglomerations were also observed in the SEM images due to the presence of particle agglomerations, an exact value of particle size was not easy to calculate. To get further more evidence on structural information of the synthesized La_2_O_3_ NPs, TEM analysis with selected-area electron diffraction (SAED) was carried out. The TEM analysis shows the agglomerated sample in the nanometer range. The TEM micrographs of the synthesized doped (Sm^3+^, Gd^3+^ and Hf^3+^) La_2_O_3_ NPs using co-precipitation method were shown in Fig. 6a–c and the corresponding SAED pattern was shown in Fig. 6d. The TEM images reveal that the surfaces of the samples are very rough. It can be further observed that the samples consist of many even smaller nanoparticles with the size of 10–30 nm. In addition, the existence of detectable diffraction rings in the selected area electron diffraction (SAED) pattern of the La_2_O_3_ NPs, further reveal the formation of polycrystalline products. These results further confirm the formation of the La_2_O_3_ NPs.

### Catalytic activity

#### Catalytic reduction of 4-nitrophenol

**Fig. 7:**
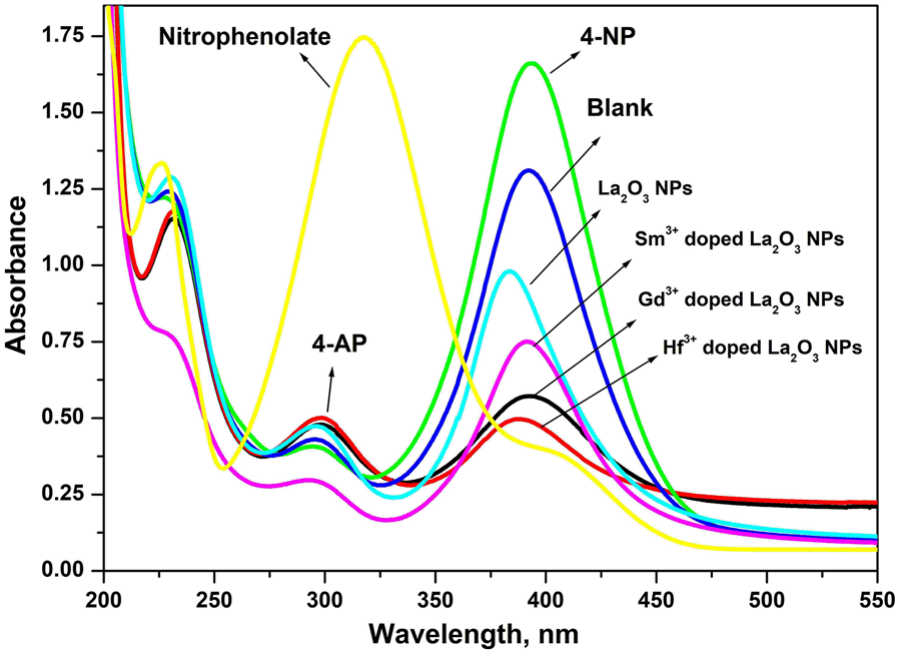
UV-Vis spectra of 4-NP reduction with undoped and doped La_2_O_3_ NPs at final time (90 min)

The catalytic activity of the as-prepared undoped and doped La_2_O_3_ NPs was confirmed through reduction of 4-nitrophenol (4-NP) in the presence of NaBH_4_ as a reducing agent. A comparison study of catalytic activities between undoped and doped (Sm^3+^, Gd^3+^ and Hf^3+^) La_2_O_3_ NPs for reduction of 4-NP was also investigated. Firstly, the 4-NP reduction has been carried out without a catalyst, it gives approximately 9% of conversion from 4-NP to 4-AP. After the addition of undoped La_2_O_3_ NPs as a catalyst, the reduction of 4-NP started immediately, and the color of the reaction solution became lighter, resulted in approximately 98% conversion. The catalytic reduction reaction of 4-NP using undoped and doped La_2_O_3_ NPs was studied by UV–vis spectrophotometrically and presented in Fig. 7. At the initial time, (t = 0 min; A_0_), the UV–vis spectra of 4-NP were characterized by the presence of a sharp band at 400 nm, owing to the formation of the nitrophenolate ion. Addition of La_2_O_3_ NPs to the reaction medium accounted for the rapid decline in the absorption intensity at 400 nm simultaneously accompanied by the appearance of a relatively wider band at 298 nm indicating the formation of 4-AP [2, 39] and 4-NP was completely reduced 4-AP within the 30 min of the reaction. The results indicate different magnitude for decreasing of absorbance at 400 nm obtained by each kind of catalyst with progressed time. The faster diminish of this peak, i.e. the reduction of 4-NP, revealed that one catalyst shows better catalytic activity than the others. Similarly, we investigated the catalytic activity of doped (Sm^3+^, Gd^3+^ and Hf^3+^) La_2_O_3_ NPs under similar conditions. Among the three samples, Hf^3+^ doped La_2_O_3_ NPs exhibited efficient catalytic activity for the reduction of 4-NP due to it has a small size of the particles and high surface area.

The conversion percentage (α) of 4-NP to 4-AP was calculated by the formula:

$$\alpha = {\text{A}}_{0} - {\text{A}}_{\text{t}} /{\text{A}}_{0} \times 100\%$$

The conversion percentage of 4-NP to 4-AP was calculated from the Fig. 7. It has been found that the reduction of 4-NP to 4-AP by NaBH_4_ in the presence of Hf^3+^ doped La_2_O_3_ NPs as a catalyst than the other samples.

#### The proposed mechanism of 4-nitrophenol reduction

A mechanism was proposed for the reduction of 4-NP to 4-AP using undoped and doped La_2_O_3_ NPs, which is exhibit excellent catalytic properties owing to the high rate of surface adsorption and high surface area to volume ratio. This hypothesis was subjected to test in two parallel studies. In the first approach, 4-NP was reduced to 4-AP in the presence of La_2_O_3_ NPs. Addition of NaBH_4_ to the reaction medium causes deprotonation of 4-NP resulting in the formation of intermediate nitrophenolate ion [2, 40]. Subsequently added NaBH_4_ reduces nitrophenolate ion to form 4-AP. The presence of NaBH_4_ favors the formation of 4-AP on account of the decrease in free energy (E_0_ for 4-NP/4-AP = − 0.76 V). After the addition of NaBH_4_, the La_2_O_3_ NPs start the catalytic reduction by relaying electrons from the donor NaBH_4_ to the acceptor 4-NP right after the adsorption of both onto the catalyst surface. The excess NaBH_4_ used, increases the pH of the reaction medium and thus retard the degradation of borohydride ions. The reduction of oxygen proceeds much faster than the nitrophenols present in the system. The reduction reaction of 4-NP only starts after all the oxygen in the system has reacted. The evolution of small bubbles of the hydrogen gas surrounding the catalyst particles remain well distributed in the reaction mixture during the course of the reaction and offer a favorable condition for a smooth reaction to occur. As NaBH_4_ is present in large excess, its consumption for the reduction of oxygen did not alter its concentration notably. However, the viability of reaction decreases as a result of the large potential difference between donor (NaBH_4_) and acceptor molecules (nitrophenolate ion) accounting for high kinetic energy barrier.

#### Evaluation of rate constants

**Fig. 8:**
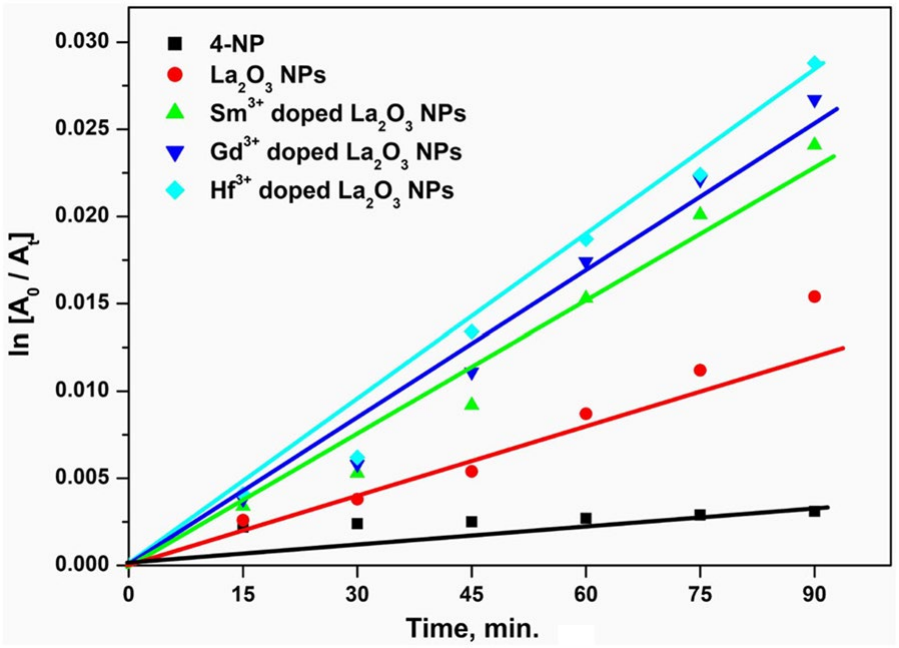
Linear plots of ln(A_0_/A_t_) vs. time for catalytic reduction of 4-nitrophenol over the synthesized undoped and doped La_2_O_3_ NPs

The reduction of 4-NP on nanoparticles has been demonstrated to follow the Langmuir–Hinshelwood mechanism involving the surface adsorption of the reducing agent and the nitrophenol substrate on the catalyst [2, 41]. In order to compare the catalytic activity of undoped and doped La_2_O_3_ NPs, a linear relationship between normalized concentration (ln A_0_/A_t_) and reaction time was derived with the synthesized sample as shown in Fig. 8. The linear plot of (ln A_0_/A_t_) vs. time, obeys the pseudo-first-order kinetic model (Fig. 8) where A_0_ and A_t_ are the initial and final concentration at time t of 4-nitrophenolate respectively. The reaction rate constant k was found to be 1.854 × 10^−2^ s^−1^ for undoped La_2_O_3_ NPs, 2.657 × 10^−2^ s^−1^ for Sm^3+^ doped La_2_O_3_ NPs, 3.614 × 10^−2^ s^−1^ for Gd^3+^ doped La_2_O_3_ NPs and 4.122 × 10^−2^ s^−1^ for Hf^3+^ doped La_2_O_3_ NPs. It was observed that the Hf^3+^ doped La_2_O_3_ NPs sample exhibited the preeminent catalytic activity among the other doped and undoped La_2_O_3_ NPs.

#### Catalytic conversion of synthesized undoped and doped La_2_O_3_ NPs

**Fig. 9:**
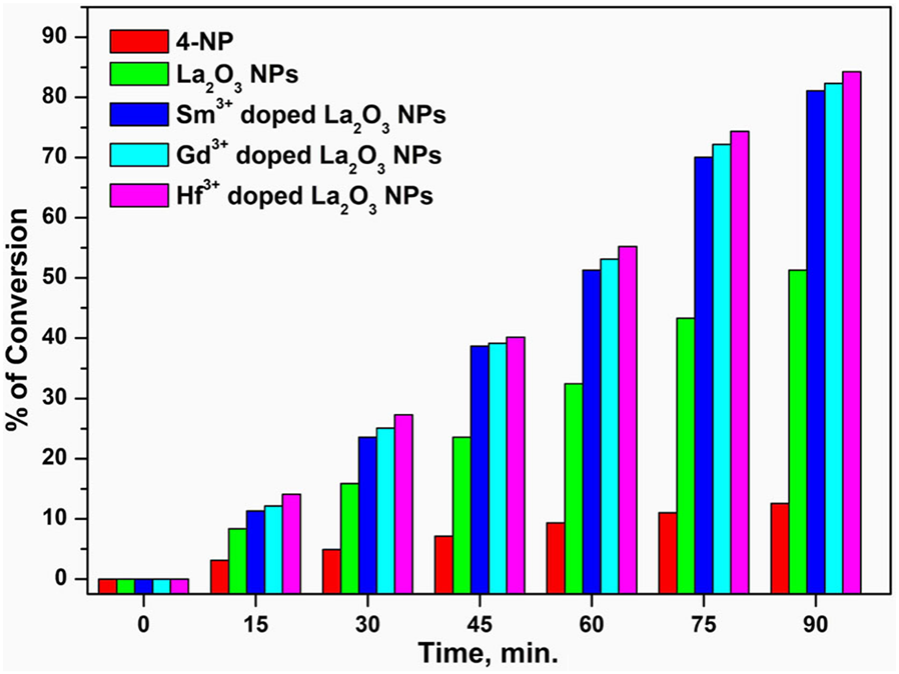
The catalytic conversion of 4-NP using undoped and doped La_2_O_3_ NPs catalyst in the presence of NaBH_4_

The estimation of catalytic conversion of 4-NP to 4-AP using the synthesized nanocatalysts is an important aspect to be strongly considered in practical heterogeneous catalytic applications. The synthesized doped and undoped La_2_O_3_ NPs were used as catalysts for the reduction of 4-NP and compared their catalytic conversion. After completion of the catalytic reactions, the percentage of conversion of the synthesized samples was measured and drawn a plot against the reaction time and shown in Fig. 9. The kinetic constant of the first run was taken as a control and the reaction conditions were kept constant for successive cycles. It was explored that the Hf^3+^ doped La_2_O_3_ NPs exhibited higher catalytic conversion in 90 min of reaction time than the other doped and undoped La_2_O_3_ NPs.

## Conclusions

In this study, we have successfully demonstrated the synthesis of undoped and doped La_2_O_3_ NPs under a facile co-precipitation method. The morphological, structural and optical properties of as-synthesized La_2_O_3_ NPs were characterized using TEM, SEM, XRD, FTIR, UV–vis, and PL spectroscopy. The XRD patterns indicate that the well-crystallized and hexagonal phase La_2_O_3_ nanocrystals can be easily obtained under the current synthetic conditions. The SEM and TEM analysis revealed the size of the NPs are in the range of 30 nm with different shapes due to some agglomeration. It also demonstrated that the synthesized undoped and doped La_2_O_3_ NPs as nanocatalysts strongly influent catalytic performance for 4-NP reduction using NaBH_4_ as a reducing agent. The catalytic activities of Hf^3+^ doped La_2_O_3_ NPs are higher than the Sm^3+^, Gd^3+^ doped and undoped La_2_O_3_ NPs due to the smaller size and high surface area. The well dispersed doped La_2_O_3_ NPs having the size 10–30 nm were exhibited the best catalytic activity for reduction of 4-NP. Our results demonstrate that the doped La_2_O_3_ NPs can be a promising material of choice for various practical applications in catalysis and industrial applications.
